# Supplementary material for: Small-molecule inhibition of Lats kinases may promote Yap-dependent proliferation in postmitotic mammalian tissues
Source: Nat Commun. 2021 May 25;12:3100. doi: 10.1038/s41467-021-23395-3 (PMC8149661; doi:10.1038/s41467-021-23395-3)
Supplement: Supplementary file 1 — Supplementary Information [file 41467_2021_23395_MOESM1_ESM.pdf]

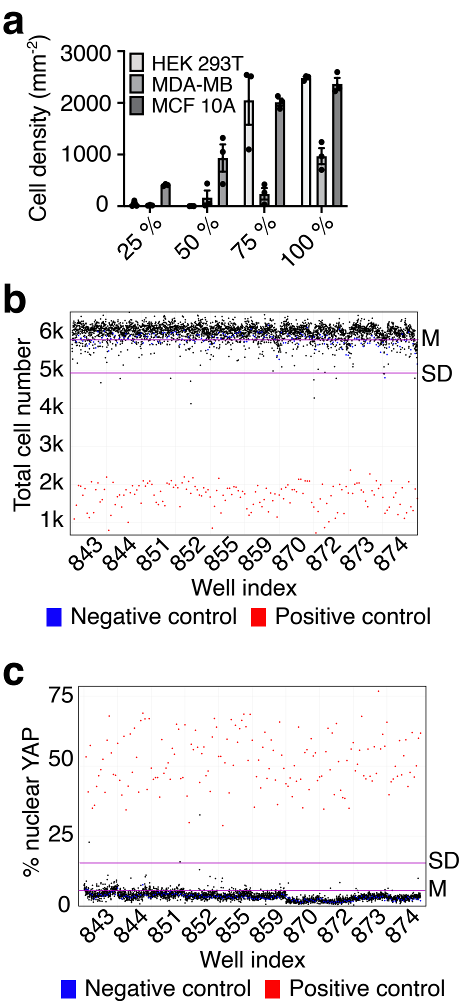

Supplementary Figure 1. Screening for activators of Yap translocation into nuclei. Related to Figure 1. (a) The adherent properties of three human epithelial-cell lines—one embryonic kidney line (HEK 293T) and two mammary-gland lines (MDA-MB and MCF 10A)—were tested. Only MCF 10A cells display a reproducible correlation between the number of cells seeded and the number of cells adhered after 24 hr (n = 3 for each condition, mean ± SEM). (b) In a representative example of cell survival in ten 384-well plates screened in the assay for nuclear translocation of Yap, the plate indexes are indicated on the x-axis and the total number of cells detected in each well of each plate is indicated on the y-axis. Purple lines demonstrate the average mean (M) and one standard deviation (SD) below the mean of the number of cells detected in each negative control well (blue dots) of each of the ten plates screened. Red dots represent sub-confluent positive control wells; black dots represent each well treated with a compound. The substances that decreased the number of cells over one SD were eliminated owing to toxicity. (c) In the same ten 384-well plates analyzed in panel B, the percentage of cells with nuclear Yap was determined using MetaXpress software. Only one in a thousand compounds increased this value more than one standard deviation (SD; purple line) above the mean value determined for the negative controls (M; purple line).

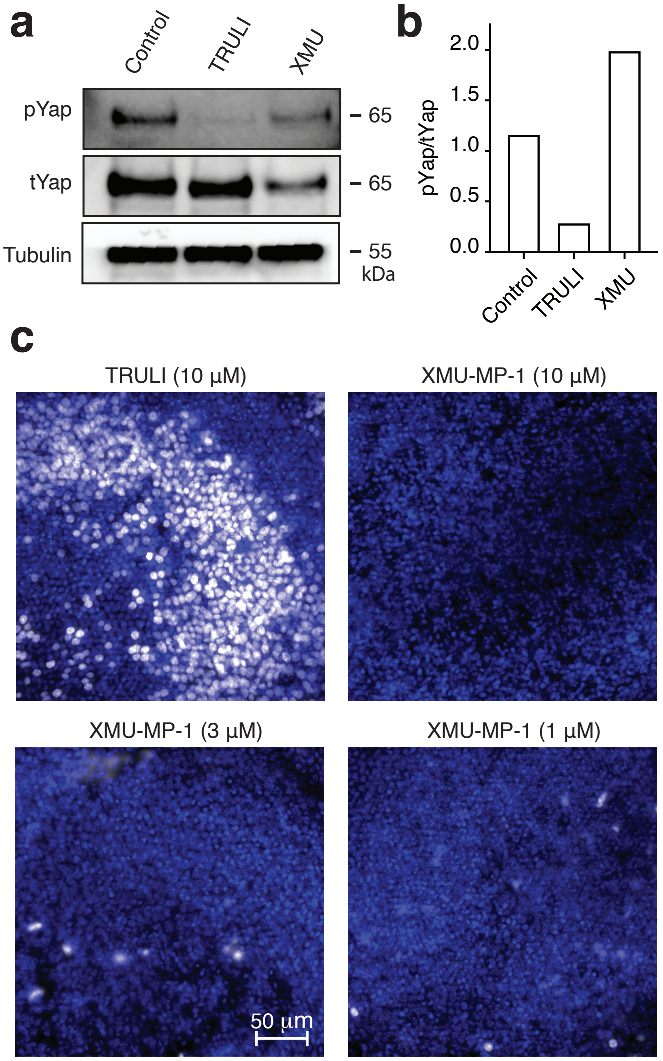

Supplementary Figure 2. Lack of Yap activation by the Mst inhibitor XMU-MP-1. Related to Figure 2. (A) Six utricles per condition were treated for 24 hr with 10 µM TRULI or XMU-MP-1 to assess effects on the amount of phospho-Yap by comparison to control conditions. (B) Quantification of band-intensity ratios for phospho-Yap and total Yap in the immunoblot of panel A shows that XMU-MP-1 does not reduce the fraction of phosphorylated Yap. (C) When adult mouse utricles were explanted and cultured with EdU (white) and either TRULI or varying concentrations of XMU-MP-1, the latter caused no increase in proliferation. Nuclei are stained with DAPI (blue). This assay was repeated at least twice with the same results.

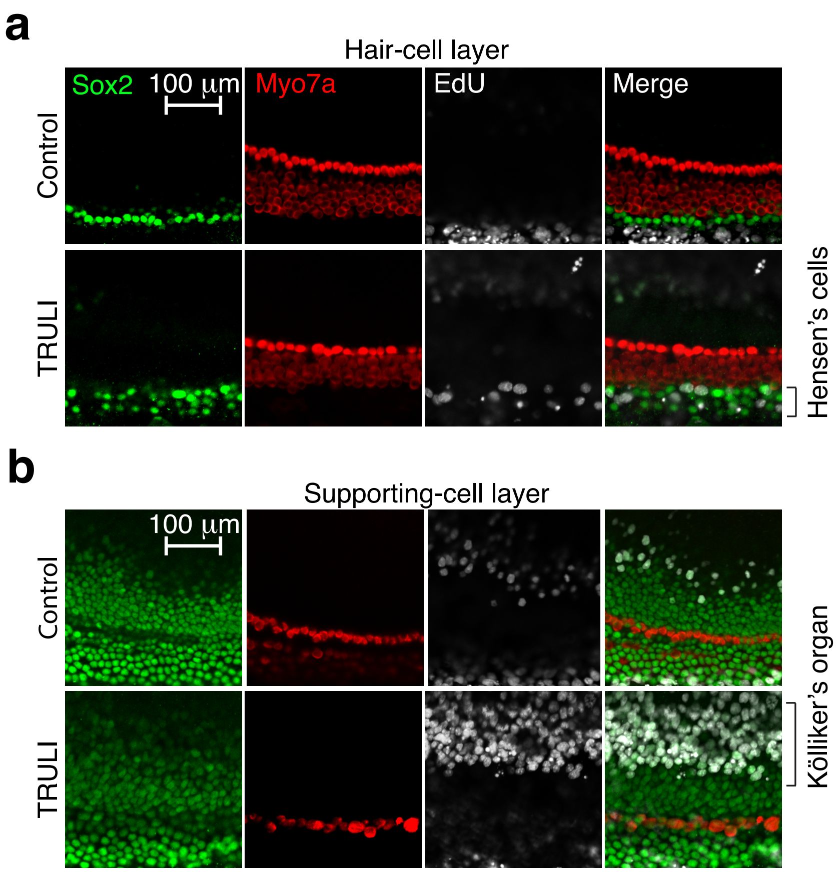

Supplementary Figure 3. Proliferative response in the cochlea after treatment with TRULI. Related to Figure 2. (a) No hair-cell loss is observed after 5 d of treatment of the P1 organ with 10 μM TRULI (n = 6). Only the most lateral rows of Hensen's cells re-enter the cell cycle after the treatment. (b) TRULI elicits robust proliferation of Sox2-positive cells in Kölliker's organ, as measured by the incorporation of EdU. This assay was repeated three times on two explants each time with the same results.

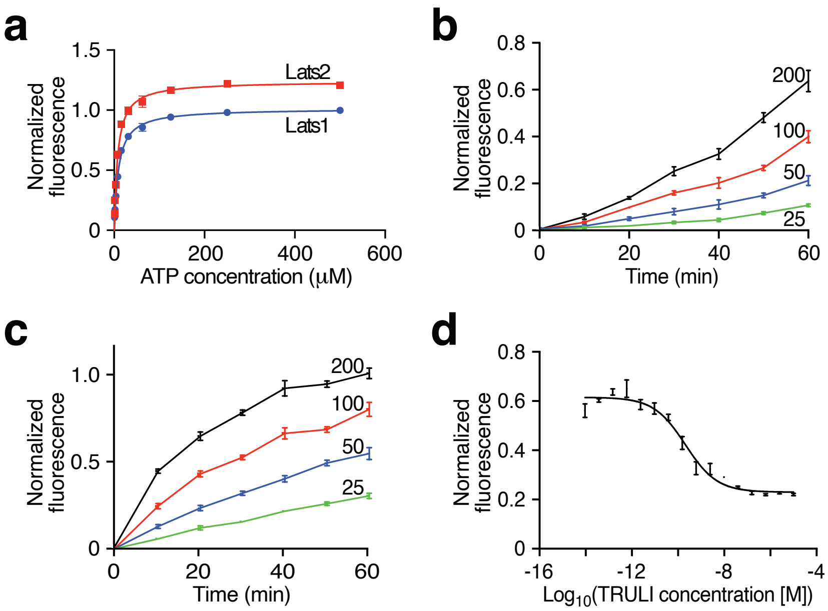

Supplementary Figure 4. Optimization and further results of *in vitro* kinase assay. Related to Figure 3. (A) In order to perform assays at an appropriate ATP concentration, we established the Michaelis-Menten constants for Lats1 (K_m = 9.4 μM) and Lats2 (K_m = 7.6 μM). (B) A kinetic assay for Lats1 was used to determine the optimal signal-to-noise ratio within the linear range of product formation. Enzyme concentrations are indicated in micrograms per liter. (C) The corresponding assay was performed for Lats2. (D) The half-maximal inhibitory concentration of TRULI against Lats2 was IC_{50} = 0.2 nM.

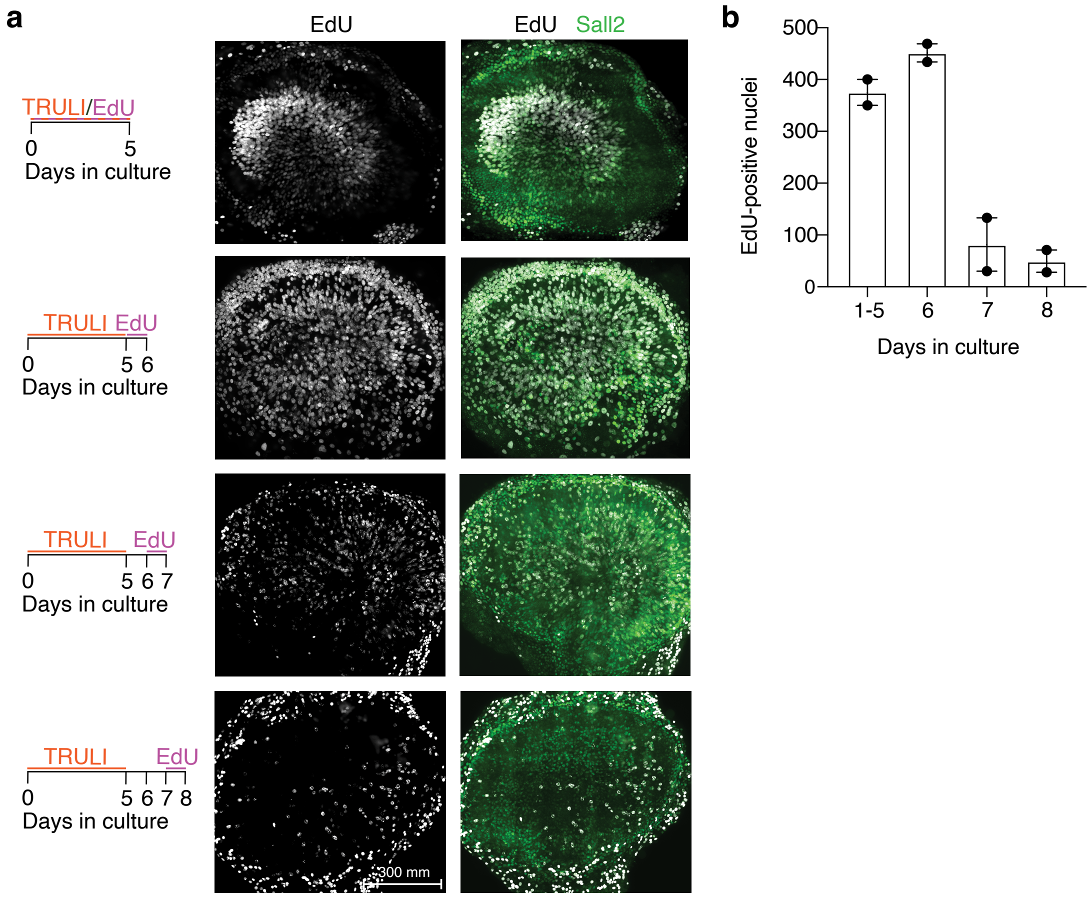

Supplementary Figure 5. Cell-cycle exit after TRULI withdrawal. Related to Figure 4. (A) As shown in the timelines to the left, utricles were explanted and cultured for 5 d in the presence of TRULI; EdU was then added at various times. In first top row, when EdU was present throughout the 5 d period of culture, extensive proliferation occurred in the striolar region. The second row shows that proliferation was still heavier when EdU was presented during the day following TRULI exposure. The third row indicates that proliferation declined during the second day after TRULI withdrawal. The fourth row demonstrates that proliferation fell to nearly the background level when EdU was provided three days after the withdrawal of TRULI. (B) A bar graph quantifies the number of EdU-positive nuclei per sensory epithelium in the foregoing experiments. Each datum is an average from two utricles.

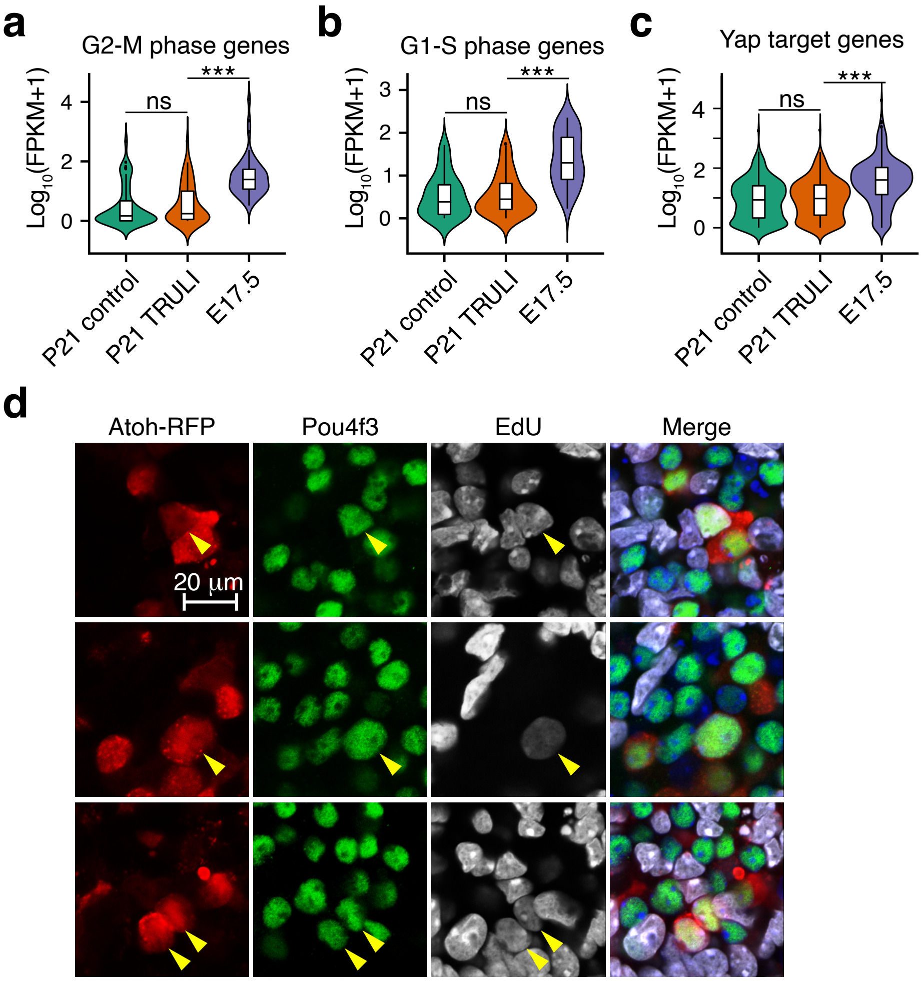

Supplementary Figure 6. Cell-cycle exit and hair cell differentiation after TRULI withdrawal. Related to Figure 5. (A) RNA-sequencing analysis demonstrates that the genes active in G2-M are downregulated to the control levels in P21 supporting cells 5 d after TRULI withdrawal similarly downregulated to the DMSO-control levels (p = 0.2908 by a Wilcoxon rank sum test with continuity correction, n = 52 genes for each condition) and are significantly lower than that found in E17.5 supporting cells ($p = 3.443 \cdot 10^{-9}$ by a two-sided Wilcoxon rank sum test with continuity correction, n = 52 genes for each condition). Box plots indicate median (middle line), 25th and 75th percentile (box) and 5th and 95th percentile (whiskers) as well as outliers (single points). (B) S-phase genes are similarly downregulated to the DMSO-control levels (p = 0.7036 by a two-sided Wilcoxon rank sum test with continuity correction, n = 42 genes for each condition) and are significantly lower than that found in E17.5 supporting cells (p = 1.526e-08 by a Wilcoxon rank sum test with continuity correction, n = 42 genes for each condition). Box plots indicate median (middle line), 25th and 75th percentile (box) and 5th and 95th percentile (whiskers) as well as outliers (single points). (C) Genes known to be targets of theYap-Tead complex are also downregulated to the DMSO-control levels (p = 0.3447 by a Wilcoxon rank sum test with continuity correction, n = 337 genes for each condition) and are significantly lower than that found in E17.5 supporting cells ($p = 2.2 \cdot 10^{-16}$ by a Wilcoxon rank sum test with continuity correction, n = 337 genes for

each condition). Box plots indicate median (middle line), 25th and 75th percentile (box) and 5th and 95th percentile (whiskers) as well as outliers (single points). (D) To assess whether new hair cells can be formed after TRULI treatment, P21 utricles that had been treated with TRULI for 5 d were cultured for two days to allow cell-cycle exit, then transfected with adenovirus carrying Atoh1. The cultures were co-treated with EdU to permit lineage tracing of supporting cells that had undergone cell division in the presence of TRULI. After 5 d of Atoh1 expression, many EdU-positive supporting cells displayed high levels of Pou4f3 protein, demonstrating the potential of supporting cells to transdifferentiate. The experiment was repeated 3 times (n = 2 utricles each time) with similar results.

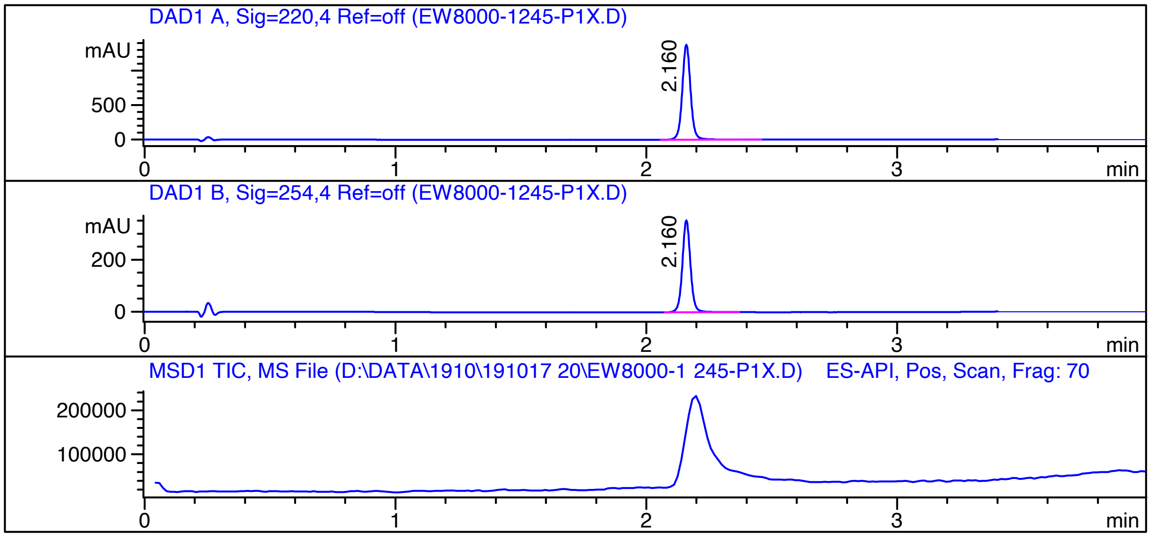

Supplementary Figure 7. Liquid chromatography–mass spectrometry (LC–MS) of TRULI.
MS (m/z): $[M]^+$ calculated for $C_{18}H_{14}N_4OS$, 334.09; found, 335.1 $[M + H]^+$.

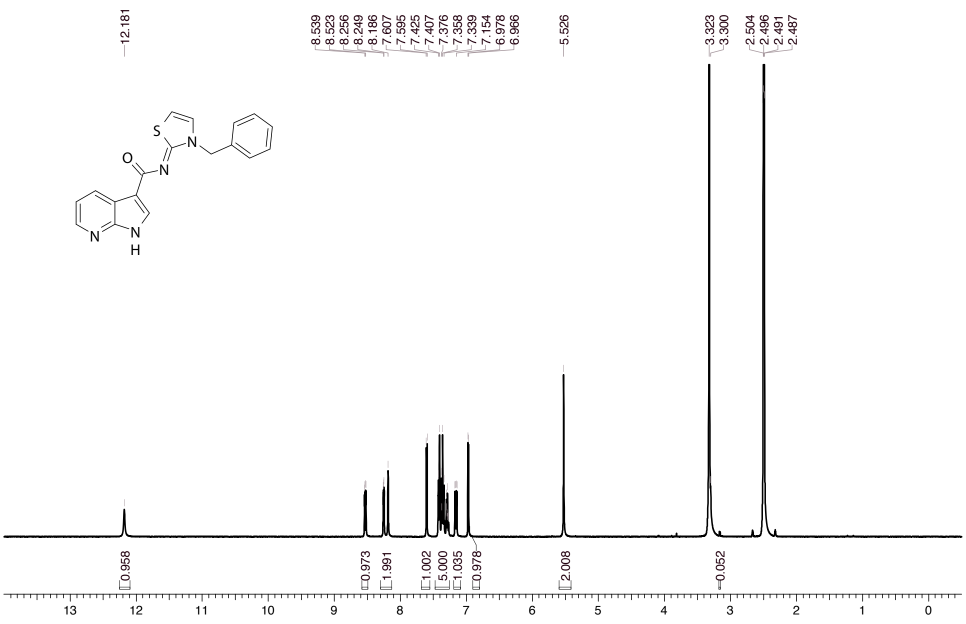

Acquisition Time (sec)	3.0671
Comment	EW8000-1 245-P1B1 DMSO Bruker_E_ 400MHz
Date	17 Oct 2019 09:39:13
Frequency (MHz)	400.1500
Nucleus	1H
Number of Transients	8
Origin	spect
Original Points Count	24576
Owner	nmrsu
Points Count	65536
Pulse Sequence	zg30
Receiver Gain	160.91
SW(cyclical) (Hz)	8012.82
Solvent	DMSO-d6
Spectrum Offset (Hz)	2395.9778
Spectrum Type	standard
Sweep Width (Hz)	8012.70
Temperature (degree C)	24.744

Supplementary Figure 8. Nuclear magnetic resonance (NMR) of TRULI. ^{1}H NMR (DMSO, 400 MHz): δ 12.18 (br. s, 1H), 8.53-8.50(m, 1H), 8.25-8.10 (m, 2H), 7.60 (d, J = 4.8 Hz, 1H), 7.42 - 7.40 (m, 5H), 7.36 (d, J = 7.2 Hz, 1H), 6.96 (d, J = 4.8 Hz, 1H), 5.52 (s, 2H).

Supplementary Table 1. Alignment of residues at the ATP-binding sites of AGC kinases. Related to Figure 3. These 36 residues define the ATP-binding pockets of 62 kinases, but do not represent continuous sequences.

LATS1	LGIGAFGEVAKVRLFVMDYIPGGDMMSDKDNLTDFG
LATS2	LGIGAFGEVAKVKLFVMDYIPGGDMMSDKDNLTDFG
NDR1	IGRGAFGEVAKVKMLIMEFLPGGDMMTDKDNLSDFG
NDR2	IGRGAFGEVAKVKMLIMEFLPGGDMMTDKDNLSDFG
ROCK1	IGRGAFGEVAKVQLMVMEYMPGGDLVNDKDNLADFG
ROCK2	IGRGAFGEVAKVQLMVMEYMPGGDLVNDKDNLADFG
RSKL2	VQDPATGGTMKSSGPHLNLLTPARLPSDHGNLTYFG
SgK494	VAKGSFGTVAKHSLIMCSYCSTDLYSLDKENLTDFG
PDK1	LGEGSFSTVAKVKLFGLSYAKNGELLKDKENLTDFG
RSK4	LGQGSFGKVAKVKLLILDFLRGGDVFTDKENLTDFG
RSK1	LGQGSYGKVAKVKLLILDFLRGGDLFTDKENLTDFG
RSK3	LGQGSFGKVAKVKLLILDFLRGGDLFTDKENLTDFG
RSK2	LGQGSFGKVAKVKLLILDFLRGGDLFTDKENLTDFG
p70S6K	LGKGGYGKVAKVDLLILEYLSGGELFMDKENMTDFG
p70S6Kb	LGKGGYGKVAKVELLILECLSGGELFTDKENMTDFG
MSK1	LGTGAYGKVAKVTLLILDYINGGELFTDKENLTDFG
MSK2	LGTGAYGKVAKVTLLILDYVSGGEMFTDKENLTDFG
YANK2	IGKGSFGKVAKVNLMVVDLLLGGDLRYDKDNLTDFN
YANK3	IGKGSFGKVAKVNLMVVDLLLGGDLRYDKDNLTDFN
YANK1	IGKGSFGKVAKVNLMVVDLLLGGDLRYDKDNLTDFN
PKCi	IGRGSYAKVAKVGLFVIEYVNGGDLMFDKDNLTDYG
PKCz	IGRGSYAKVAKVGLLVIEYVNGGDLMFDKDNLTDYG
SGK2	IGKGNYGKVAKVGLFVLDYVNGGELFFDKENLTDFG
SGK	IGKGSFGKVAKVGLFVLDYINGGELFYDKENLTDFG
SGK3	IGKGSFGKVAKVGLFVLDFVNGGELFFDKENLTDFG
PKN3	LGRGHFGKVAKLSLFVTEFVPGGDLMMDKDNLADFG
PKN2	LGRGHFGKVAKVNLFVMEYAAGGDLMMDKDNLADFG
PKN1	LGRGHFGKVAKVNLFVMEYSAGGDLMLDKDNLADFG
AKT1	LGKGTFGKVAKTALFVMEYANGGELFFDKENMTDFG
AKT2	LGKGTFGKVAKTALFVMEYANGGELFFDKENMTDFG
AKT3	LGKGTFGKVAKTSLFVMEYVNGGELFFDKENMTDFG
PKCt	LGKGSFGKVAKTHMFVMEYLNGGDLMYDKDNLADFG
PKCd	LGKGSFGKVAKTHLFVMEFLNGGDLMYDKDNLADFG
PKCe	LGKGSFGKVAKTQLFVMEYVNGGDLMFDKDNLADFG
PKCh	LGKGSFGKVAKTQLFVMEFVNGGDLMFDKDNLADFG
PKCg	LGKGSFGKVAKTQLFVMEYVTGGDLMYDKDNMTDFG
PKCb	LGKGSFGKVAKTQLFVMEYVNGGDLMYDKDNMADFG
PKCa	LGKGSFGKVAKTQLFVMEYVNGGDLMYDKDNMADFG
BARK2	IGRGGFGEVAKVCMFILDLMNGGDLHYDKANLSDLG
BARK1	IGRGGFGEVAKVCMFILDLMNGGDLHYDKANLSDLG
RHOK	LGKGGFGEVAKVSLLVMTIMNGGDIRYDKENLSDLG
GPRK7	LGKGGFGEVAKVSLLVMSLMNGGDLKFDKENLSDLG
GPRK6	LGKGGFGEVAKVSLLVLTLMNGGDLKFDKENLSDLG

GPRK5	LGKGGFGEVAKVNLLVLTIMNGGDLKFDKENLSDLG
GPRK4	LGKGGFGEVAKVSLLVLTIMNGGDLKFDKENLSDLG
MAST3	ISNGAYGAVAKVSMMVMEYVEGGDCATDKDNLTDFG
MAST4	ISNGAYGAVAKVSMMVMEYVEGGDCATDKDNLTDFG
MAST2	ISNGAYGAVAKVSMMVMEYVEGGDCATDKDNLTDFG
MAST1	ISNGAYGAVAKVGMMVMEYVEGGDCATDKDNLTDFG
CRIK	VGCGHFAEVAKPQLLVMEYQPGGDLLSDKENLVDFG
MASTL	ISRGAFGKVAKVHLLVMEYLIGGDVKSDKDNLTDFG
DMPK1	IGRGAFSEVAKTQLLVMEYYVGGDLLTDKDNLADFG
MRCKb	IGRGAFGEVAKTALLVMDYYVGGDLLTDKDNLADFG
DMPK2	IGRGAFGEVAKTTLLVMDYYAGGDLLTDKDNLADFG
MRCKa	IGRGAFGEVAKTTLLVMDYYVGGDLLTDKDNLADFG
PKG2	LGVGGFGRVAKVKLMLLEACLGGELWSDKENIVDFG
PKG1	LGVGGFGRVAKVRLMLMEACLGGELWTDKENIVDFG
PRKX	VGTGTFGRVAKIRLMLMEYVPGGELFSDKENLTDFG
PRKY	MGTGTFGRVAKIRLMLMEYVPGGELFSDKENLTDFG
PKACg	LGMGSFGRVAKVKLLVMEYVPGGEMFSDKENLTDFG
PKACb	LGTGSFGRVAKVRLMVMEYVPGGEMFSDKENLTDFG
PKACa	LGTGSFGRVAKVKLMVMEYVPGGEMFSDKENLTDFG

Supplementary Table 2. Percentage sequence identity for the 36 residues of the ATP-binding site between Lats1 and Lats2 and AGC kinases. Related to Figure 3. In this and the subsequent two tables, colors identify different sub-families of the AGC kinase family._Green- PKA family members, Orange- PKC family members, Blue- NDR family members, Yellow- ROCK family members

AGC kinase	Identity to Lats1 (%)	Identity to Lats2 (%)
LATS1	100.0	97.2
LATS2	97.2	100.0
PKACb	75.0	72.2
PKN2	72.2	72.2
PKN1	72.2	72.2
ROCK1	72.2	72.2
ROCK2	72.2	72.2
PKACg	72.2	75.0
PKACa	72.2	75.0
PKCe	69.4	69.4
PKCg	69.4	69.4
NDR1	69.4	72.2
NDR2	69.4	72.2
MASTL	69.4	69.4
MRCKb	69.4	69.4
DMPK2	69.4	69.4
MRCKa	69.4	69.4
SGK	66.7	66.7
PKN3	66.7	66.7
PKCt	66.7	66.7
PKCd	66.7	66.7
PKCh	66.7	66.7
PKCb	66.7	66.7
PKCa	66.7	66.7
MSK1	63.9	63.9
MSK2	63.9	63.9
CRIK	63.9	63.9
DMPK1	63.9	63.9
PRKX	63.9	61.1
PRKY	63.9	61.1
RSK4	61.1	63.9
RSK3	61.1	63.9
RSK2	61.1	63.9
YANK2	61.1	61.1
YANK3	61.1	61.1
YANK1	61.1	61.1
PKCi	61.1	61.1
SGK2	61.1	61.1
SGK3	61.1	61.1
AKT1	61.1	61.1
AKT2	61.1	61.1
AKT3	61.1	61.1
RHOK	61.1	61.1
GPRK7	61.1	61.1
MAST3	61.1	61.1
MAST4	61.1	61.1
MAST2	61.1	61.1
MAST1	61.1	61.1
RSK1	58.3	61.1
PKCz	58.3	58.3
GPRK6	58.3	58.3
GPRK5	58.3	58.3
GPRK4	58.3	58.3
PDK1	55.6	58.3
BARK2	55.6	55.6
BARK1	55.6	55.6
PKG1	55.6	52.8
p70S6K	52.8	52.8
PKG2	52.8	55.6
p70S6Kb	50.0	50.0
SgK494	44.4	44.4
RSKL2	27.8	27.8

Supplementary Table 3. Target enzymes binding more potently than LATS1. Related to Figure 3. Of 314 kinases in a binding panel, these enzymes bound 1 μM TRULI more strongly than did Lats1 or Lats2. The assay did not test for functional inhibition. Green- PKA family members, Orange- PKC family members, Blue- NDR family members, Yellow- ROCK family members.

**Kinase**	**Affinity**
CLK4	102.191
PRKCQ	99.155
CDC7	99.093
DMPK	97.919
CDC42BPA	97.161
HIPK3	95.798
HIPK2	94.205
PRKACB	92.922
CLK2	92.819
GSK3B	92.698
PRKCH	90.416
CDK19	90.205
PKN2	89.660
CDK8	88.778
PRKX	88.131
BMP2K	86.274
HIPK1	86.064
DYRK1A	85.826
PASK	85.771
ROCK2	84.111
CDC42BPG	83.657
PAK4	83.440
DYRK1B	81.514
TGFBR2	81.311
LRRK2	81.277
ROCK1	79.232
PRKG2	79.109
CLK1	78.062
PRKCD	77.995
PKN1	77.735
MAP4K4	77.535
DAPK3	77.096
TAOK1	76.404

LATS2	74.882
MAP3K7	74.047
LATS1	73.903

Supplementary Table 4. Half-maximal inhibitory concentrations (*IC_{50}*s) of TRULI for selected kinases. Related to Figure 3. Green- PKA family members, Orange- PKC family members, Blue- NDR family members, Yellow- ROCK family members.

Kinase	IC_{50} (nM)
PKA	60
PKCepsilon	14
ROCK1	88
NDR1	1000
