# Supplementary material for: Small-molecule inhibition of Lats kinases may promote Yap-dependent proliferation in postmitotic mammalian tissues
Source: Nat Commun. 2021 May 25;12:3100. doi: 10.1038/s41467-021-23395-3 (PMC8149661; doi:10.1038/s41467-021-23395-3)
Supplement: Supplementary file 3 — Description of Supplementary Files [file 41467_2021_23395_MOESM3_ESM.pdf]

Description of Additional Supplementary Files

File Name: Supplementary Data 1
Description: **Protein coding genes differentially expressed in P21 supporting cells after 5 d of TRULI treatment compared to control. Related to Figure 4.** The data generated by RNA-seq were analyzed by DESeq2. The log2FoldChanges for the differentially expressed genes were adjusted to normalize for the low counts and high dispersion values and only the protein coding genes with the |log2FoldChanges|>1 were retained. False discovery rate (FDR) was set at 0.05. The sequencing data used to generate this dataset have been deposited to NCBI's Gene Expression Omnibus (GEO) database, accession number GSE148528 [https://www.ncbi.nlm.nih.gov/geo/query/acc.cgi?acc=GSE148528].

File Name: Supplementary Data 2
Description: **Protein coding genes differentially expressed in P21 supporting cells after 5 d of TRULI treatment followed by 5 d drug withdrawal compared to control. Related to Figure 5.** The data generated by RNA-seq were analyzed by DESeq2. The log2FoldChanges for the differentially expressed genes were adjusted to normalize for the low counts and high dispersion values and only the protein coding genes with the |log2FoldChanges|>1 were retained. False discovery rate (FDR) was set at 0.05. The sequencing data used to generate this dataset have been deposited to NCBI's Gene Expression Omnibus (GEO) database, accession number GSE148528 [https://www.ncbi.nlm.nih.gov/geo/query/acc.cgi?acc=GSE148528].
