# Supplementary material for: Small-molecule inhibition of Lats kinases may promote Yap-dependent proliferation in postmitotic mammalian tissues
Source: Nat Commun. 2021 May 25;12:3100. doi: 10.1038/s41467-021-23395-3 (PMC8149661; doi:10.1038/s41467-021-23395-3)
Supplement: Supplementary file 6 — Reporting Summary [file 41467_2021_23395_MOESM6_ESM.pdf]

nature research

Corresponding author(s): Ksenia Gnedeva

Last updated by author(s): 04/09/2021

Reporting Summary

Nature Research wishes to improve the reproducibility of the work that we publish. This form provides structure for consistency and transparency in reporting. For further information on Nature Research policies, see Authors & Referees and the Editorial Policy Checklist .

Statistics

For all statistical analyses, confirm that the following items are present in the figure legend, table legend, main text, or Methods section.

n/a	Confirmed	
☐	☒	The exact sample size (n) for each experimental group/condition, given as a discrete number and unit of measurement
☐	☒	A statement on whether measurements were taken from distinct samples or whether the same sample was measured repeatedly
☐	☒	The statistical test(s) used AND whether they are one- or two-sided<br>*Only common tests should be described solely by name; describe more complex techniques in the Methods section.*
☒	☐	A description of all covariates tested
☒	☐	A description of any assumptions or corrections, such as tests of normality and adjustment for multiple comparisons
☐	☒	A full description of the statistical parameters including central tendency (e.g. means) or other basic estimates (e.g. regression coefficient) AND variation (e.g. standard deviation) or associated estimates of uncertainty (e.g. confidence intervals)
☐	☒	For null hypothesis testing, the test statistic (e.g. F, t, r) with confidence intervals, effect sizes, degrees of freedom and P value noted<br>*Give P values as exact values whenever suitable.*
☒	☐	For Bayesian analysis, information on the choice of priors and Markov chain Monte Carlo settings
☒	☐	For hierarchical and complex designs, identification of the appropriate level for tests and full reporting of outcomes
☒	☐	Estimates of effect sizes (e.g. Cohen's d, Pearson's r), indicating how they were calculated

Our web collection on statistics for biologists contains articles on many of the points above.

Software and code

Policy information about availability of computer code

Data collection	MetaExpress (Version 4.1)
Data analysis	Cell Profiler (Version 3.1.8), ImageJ (Fiji, Version 2.0)

For manuscripts utilizing custom algorithms or software that are central to the research but not yet described in published literature, software must be made available to editors/reviewers. We strongly encourage code deposition in a community repository (e.g. GitHub). See the Nature Research guidelines for submitting code & software for further information.

Data

Policy information about availability of data

All manuscripts must include a data availability statement. This statement should provide the following information, where applicable:

- Accession codes, unique identifiers, or web links for publicly available datasets
- A list of figures that have associated raw data
- A description of any restrictions on data availability

The datasets generated during the current study have been deposited to NCBI’s Gene Expression Omnibus (GEO) database, accession number GSE148528, and are publicly available [https://www.ncbi.nlm.nih.gov/geo/query/acc.cgi?acc=GSE148528]. For each Figure, raw data from cell counting, intensity measurements for the immunohistochemistry, and images whole Western Blots that include weight markers are provided in the source data file. PDB structure of ROCK1 is available at RCSB PDB 5KKS [https://www.rcsb.org/structure/5KKS]).

Field-specific reporting

Please select the one below that is the best fit for your research. If you are not sure, read the appropriate sections before making your selection.

Life sciences study design

All studies must disclose on these points even when the disclosure is negative.

Sample size	No statistical tests were performed to determine sample size before experiments. Standard N values were chosen based on experimental standards in the respective fields. The exact sample size (n) and p-value are reported for each experiment.
Data exclusions	No data was excluded.
Replication	All experiments were replicated more than once. All attempts at replications were successful.
Randomization	No subjective evaluation was part of our assays, and therefore randomization was not necessary.
Blinding	All quantification was done with software wherein the parameters were kept identical within an assay; therefore blinding was not applicable.

Reporting for specific materials, systems and methods

We require information from authors about some types of materials, experimental systems and methods used in many studies. Here, indicate whether each material, system or method listed is relevant to your study. If you are not sure if a list item applies to your research, read the appropriate section before selecting a response.

Materials & experimental systems

n/a	Involved in the study	
☐	☒	Antibodies
☐	☒	Eukaryotic cell lines
☒	☐	Palaeontology
☐	☒	Animals and other organisms
☒	☐	Human research participants
☒	☐	Clinical data

Methods

n/a	Involved in the study	
☒	☐	ChIP-seq
☒	☐	Flow cytometry
☒	☐	MRI-based neuroimaging

Antibodies

Antibodies used	anti-Sox2 (R&D AF2018), anti-myosin 7A (Proteus 25-6790), anti-Yap (SC-101199), anti-Sall2 (HPA004162), and anti-Pou4F3 (SC-81980),anti-Ki67 (275R, Cell Marque) and anti-phosphorylated histone 3 (9701, Cell Signaling) ,Anti-Sox-9 (Cell Signaling 82630), Yap (sc-101199), phospho-Yap S127 (CST 4911), Lats1 and Lats2 (Abcam, ab70565), phospho-Lats1 S909 (CST 9157), Mst1 (CST 3682), Mob1 (CST 13730), phospho-Mob1 T35 (CST 8699), tubulin (Sigma T6793), and GAPDH(Abcam ab8245),phospho-Yap at s112 (1:1000, 13008, cell signaling), Yap (1:2000, NB110-58358, Novus) and Rabbit anti GAPDH (1:8000, PLA0125, Sigma). All fluorescent secondary antibodies were used at 1:500. All HRP secondary antibodies for western blot analysis were used at 1:10,000.
Validation	Antibody validation was not performed.

Eukaryotic cell lines

Policy information about cell lines

Cell line source(s)	MCF10A cells (ATCC CRL-10317), HEK293A (Thermo R70507), WTC-11 (Coriell Institute for Medical Research, Camden, NJ), MDA MB 231(ATCC HTB-26), HEK-293T (ATCC CRL-11268)
Authentication	None
Mycoplasma contamination	Cell lines were not tested for Mycoplasma contamination
Commonly misidentified lines (See ICLAC register)	No commonly misidentified lines were used in the study.

Animals and other organisms

Policy information about studies involving animals; ARRIVE guidelines recommended for reporting animal research

Laboratory animals	Swiss Webster mice were obtained from Charles River Laboratories;Sox2-CreER, αMHC-Cre, and ROSA26-tdTomato mice were obtained from the Jackson laboratory. Yapfl/fl were described previously (Zhang et al., 2010). Gender was ignored. Mouse age was 4-8 weeks, unless otherwise indicated.

Wild animals	None
Field-collected samples	None
Ethics oversight	Institutional Animal Care and Use Committees of The Rockefeller University, the University of Southen Califiornia, and the Weizmann Institute of Science

Note that full information on the approval of the study protocol must also be provided in the manuscript.
