# Supplementary material for: Small-molecule inhibition of Lats kinases may promote Yap-dependent proliferation in postmitotic mammalian tissues
Source: Nat Commun. 2021 May 25;12:3100. doi: 10.1038/s41467-021-23395-3 (PMC8149661; doi:10.1038/s41467-021-23395-3)

Raw Western Blots

**Figure 3A**- MCF10a cell assay

Mst1
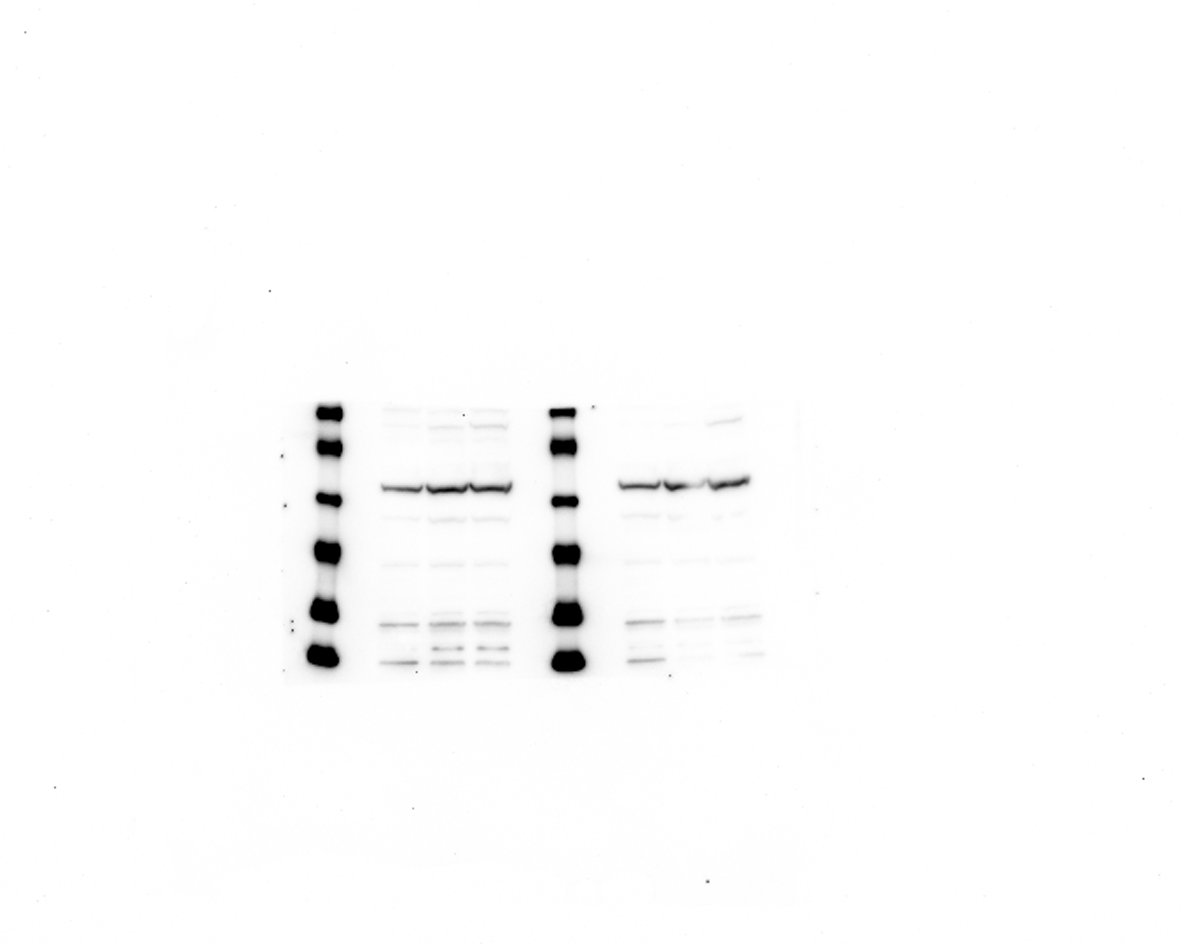


Mob1
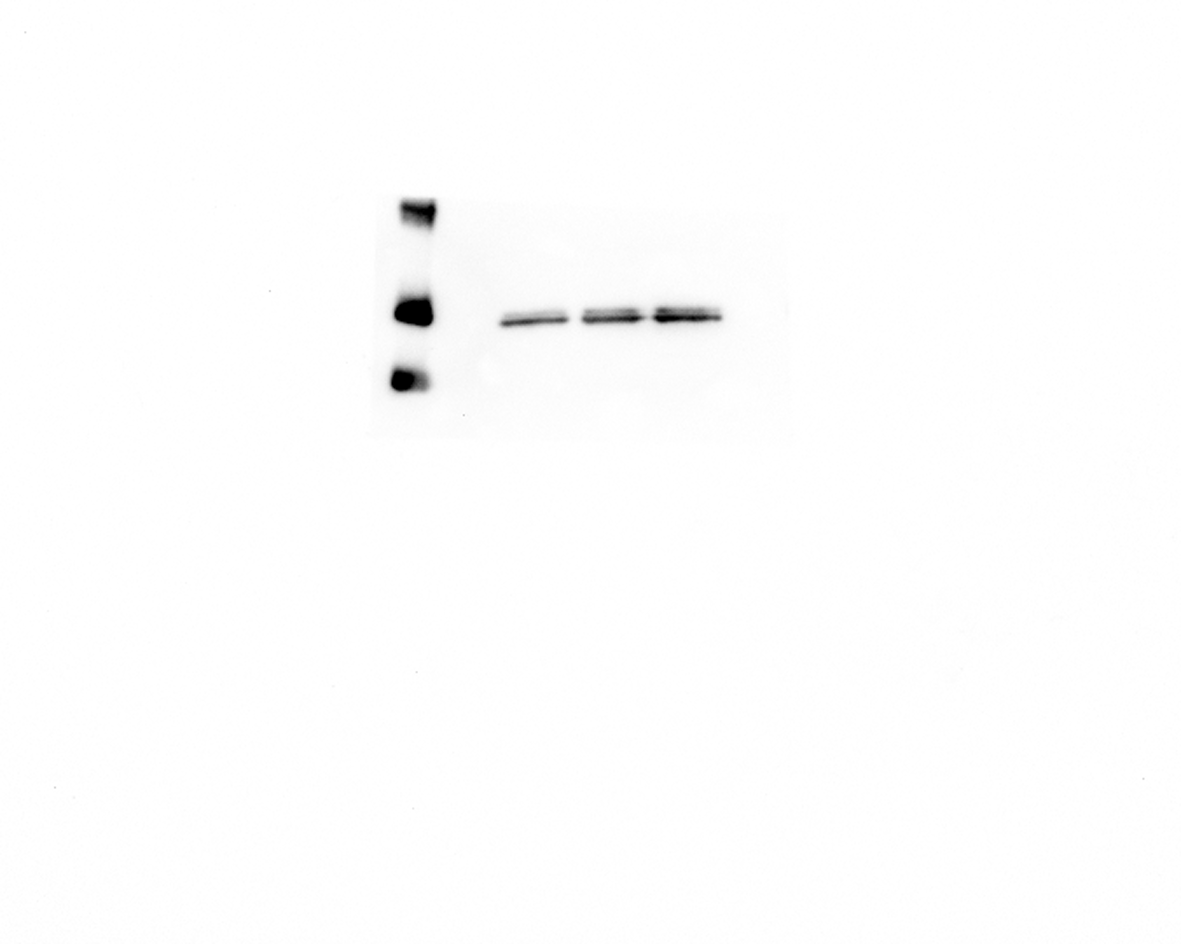


pLats_AL
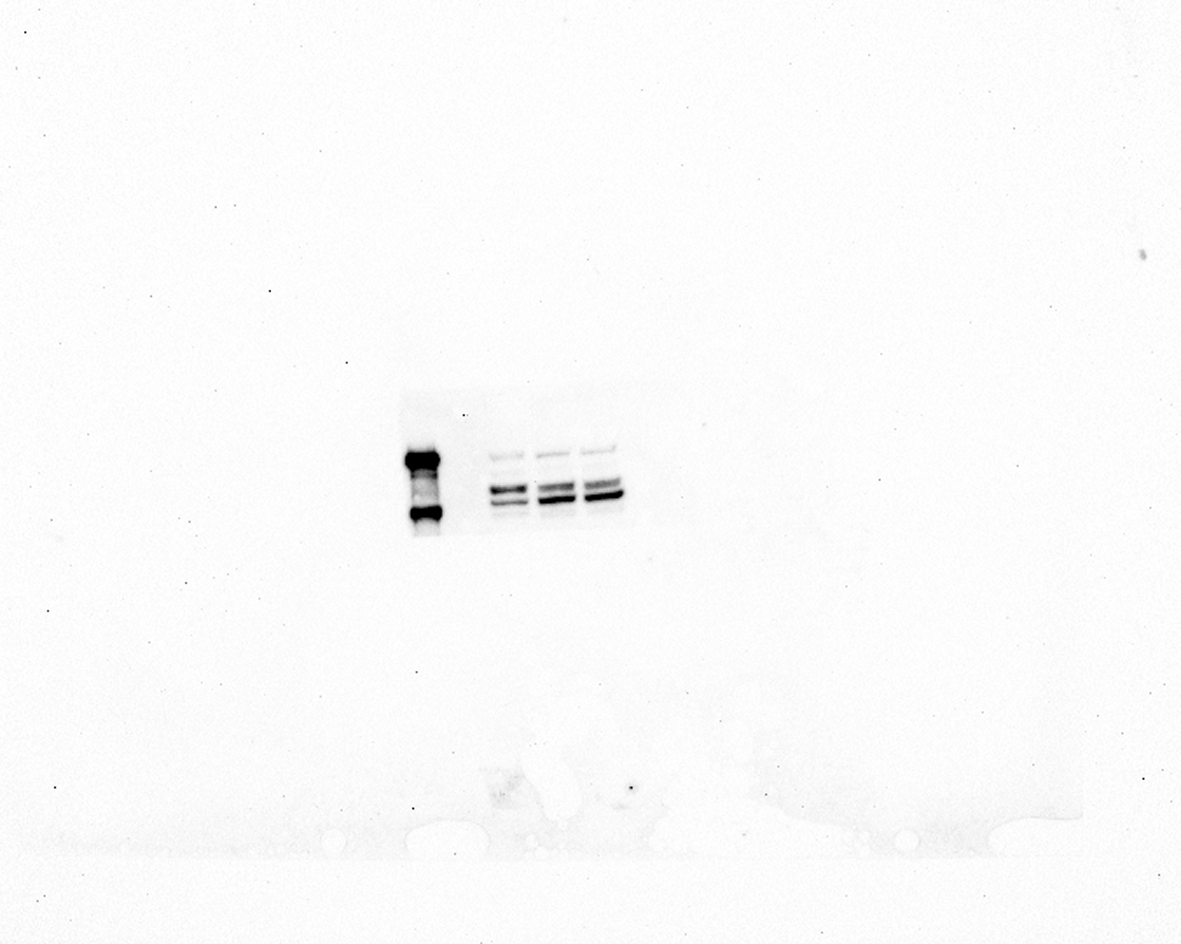


pMob1
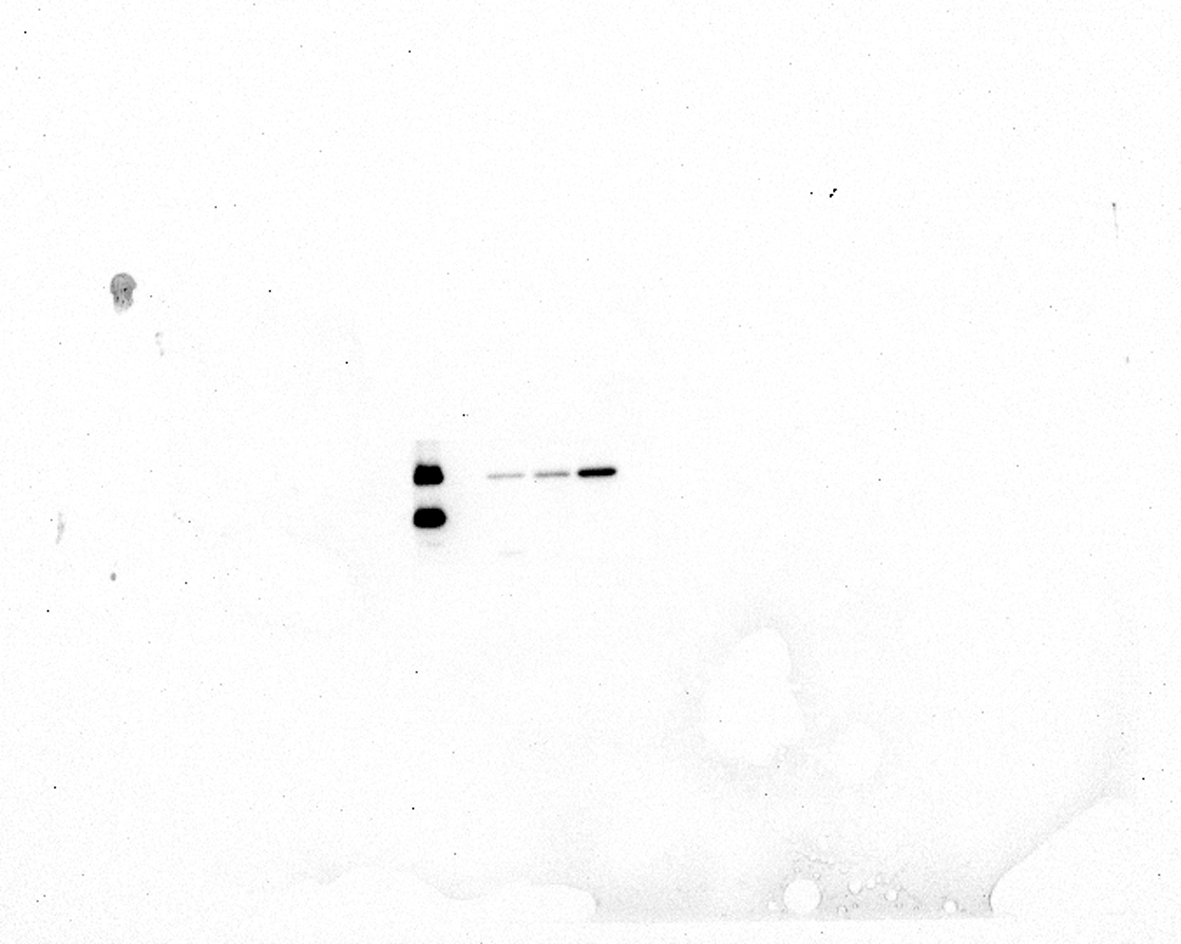


pYap
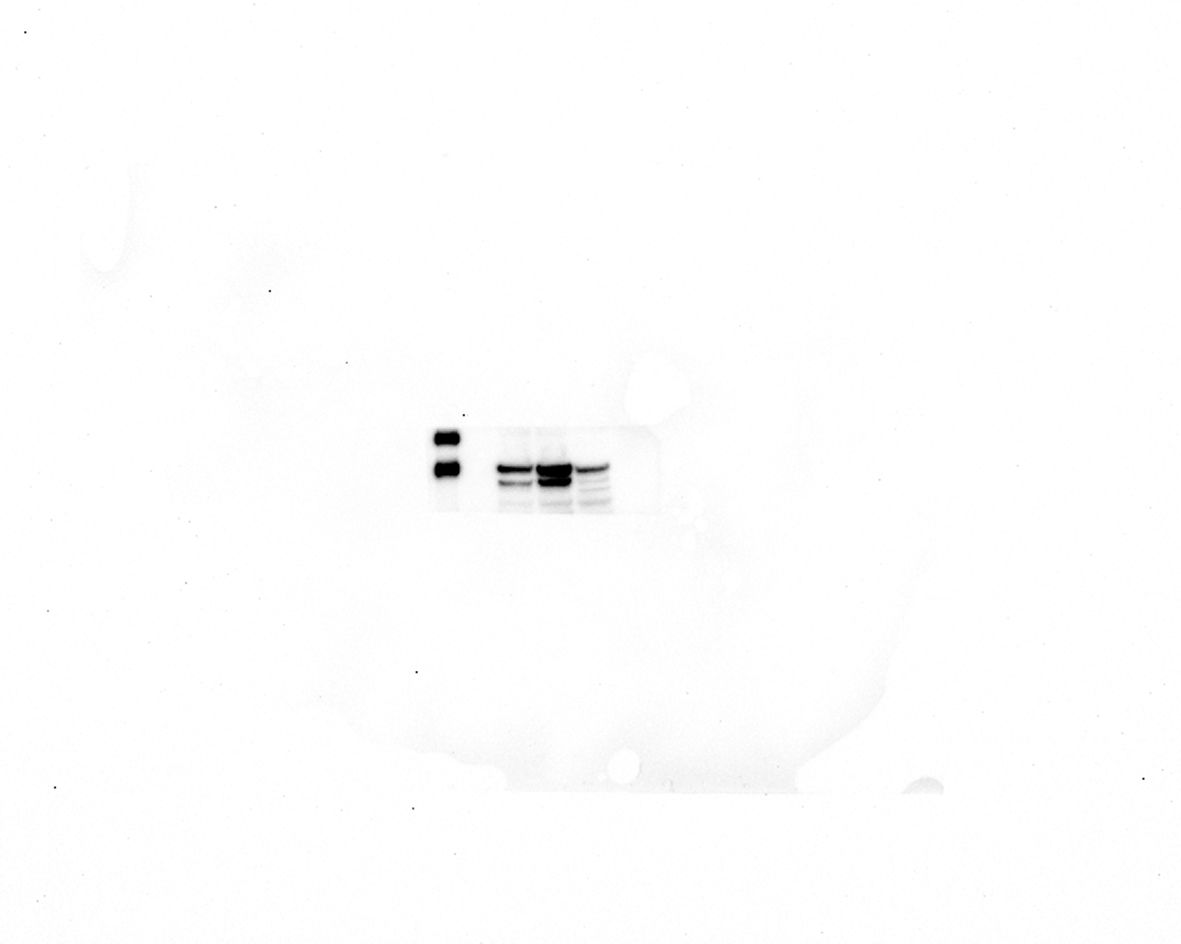


Lats1&2
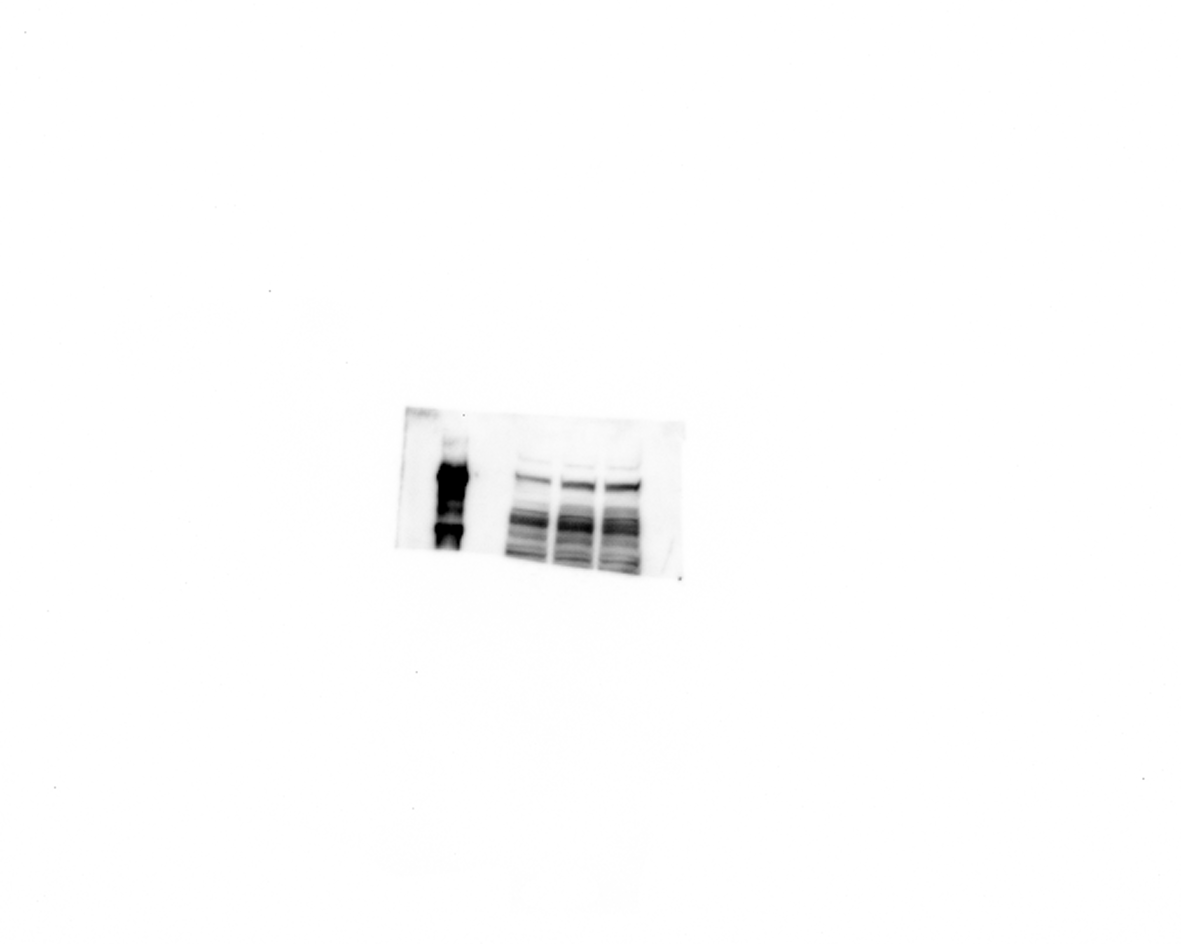


tYap
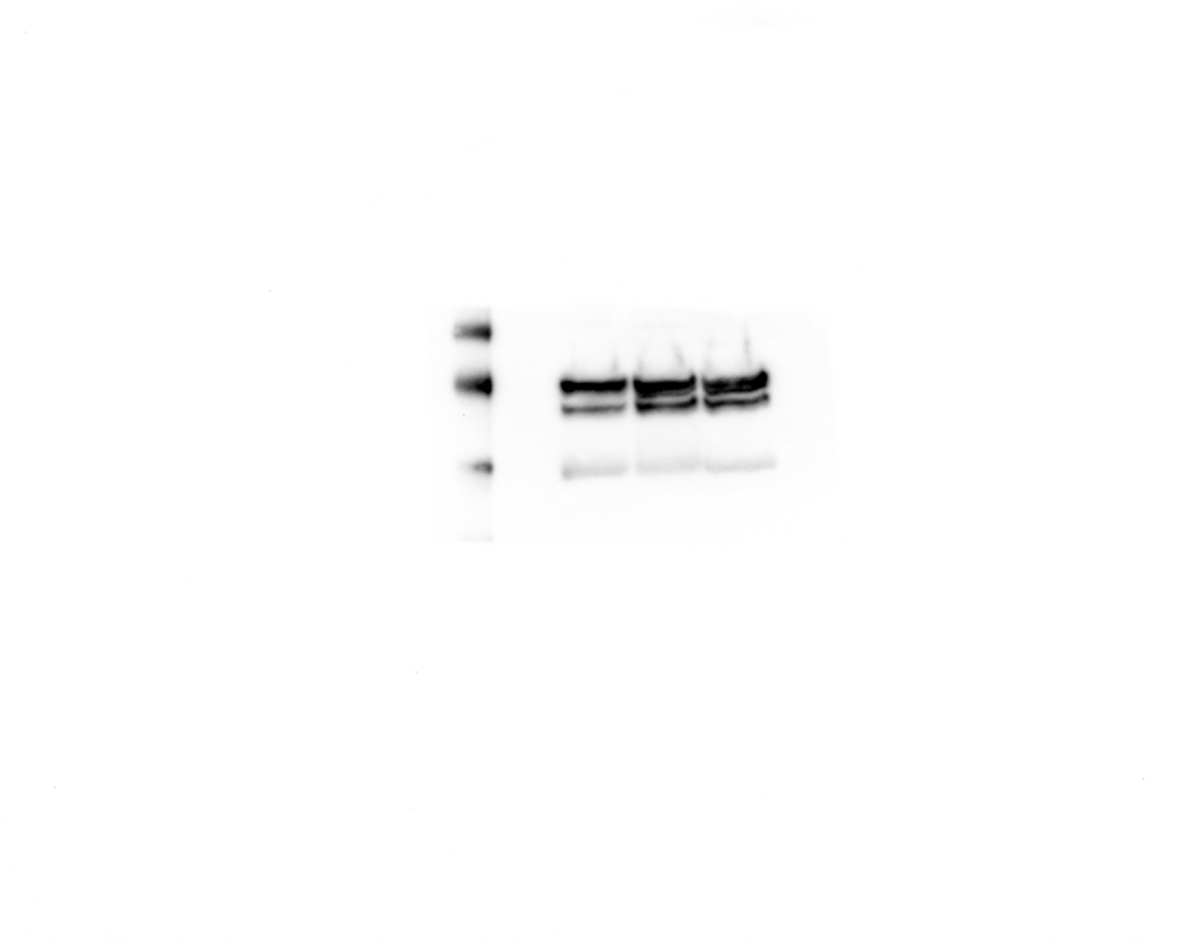


Tubulin
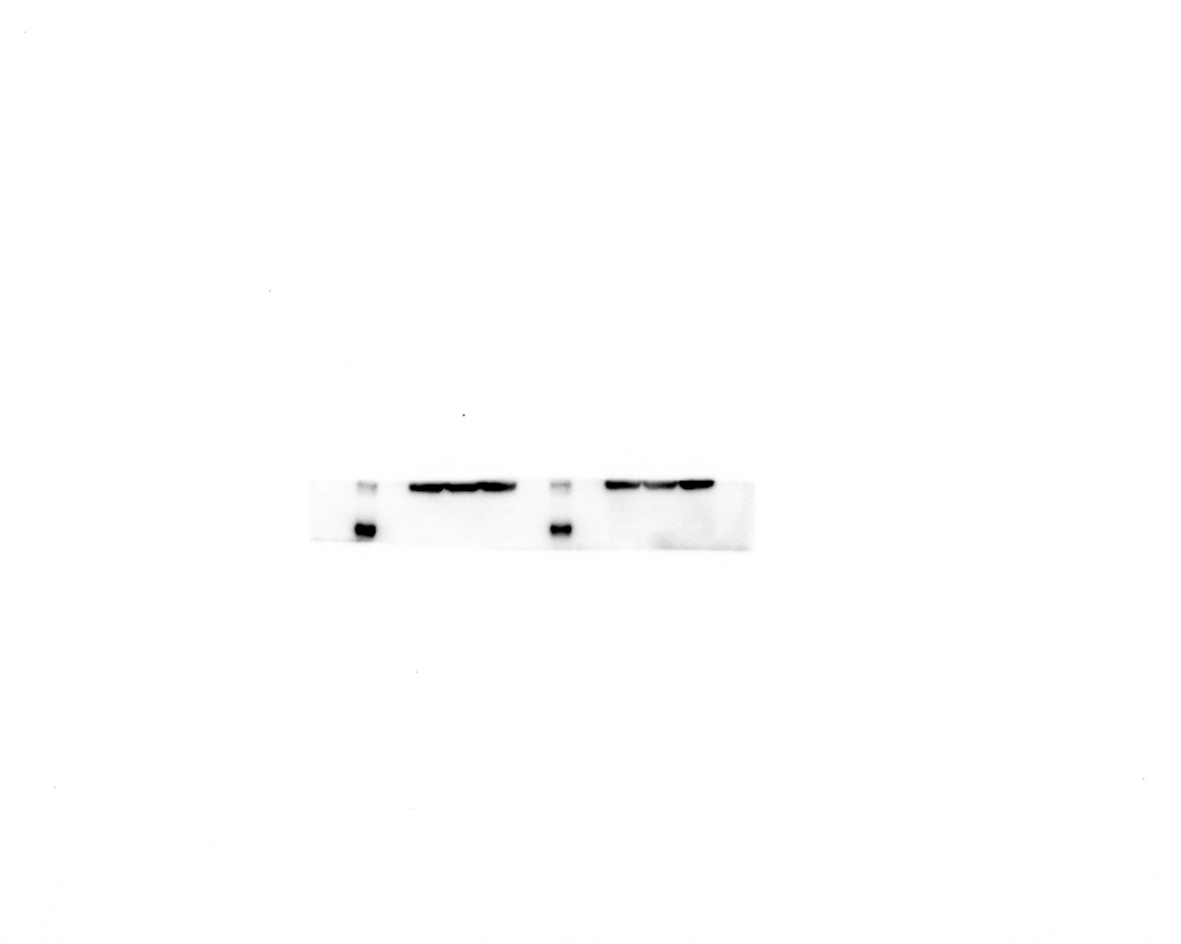


**Figure 3B** -HEK cell assay

pLats_AL

tMST

tMOB
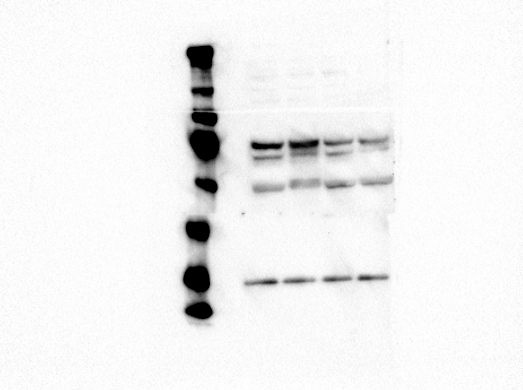


pYap
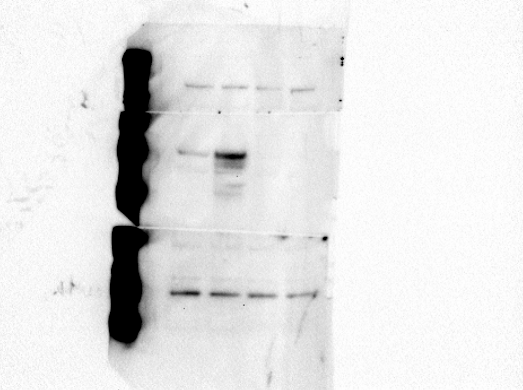


pMob1

tYap

Tubulin
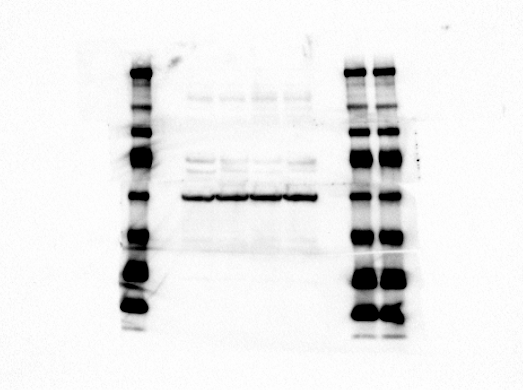


**Figure 6A** -Cardiomyocytes

pYap
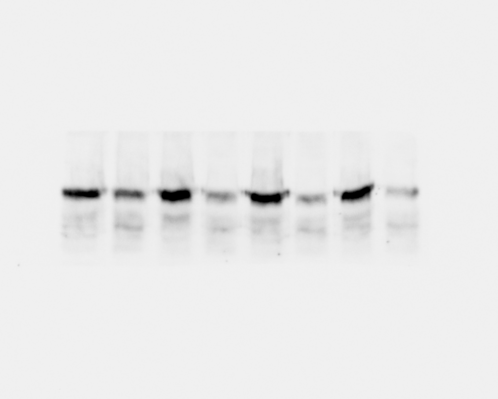


tYap
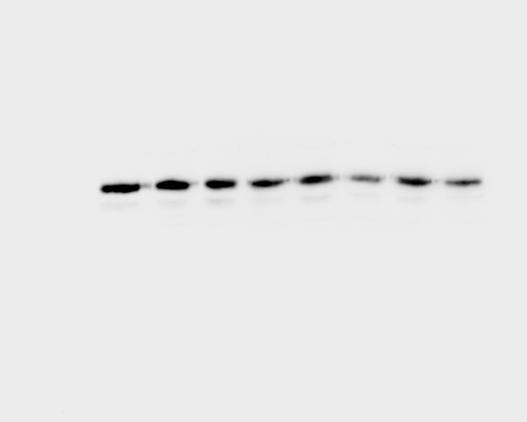


Gapdh
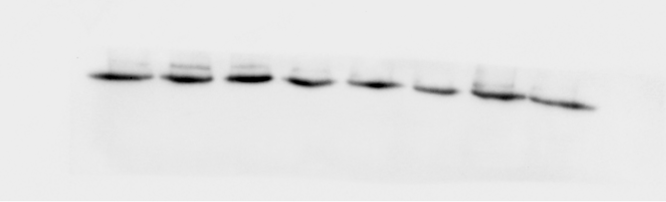


**Figure 6H**-Retinal Organoid

pYAP
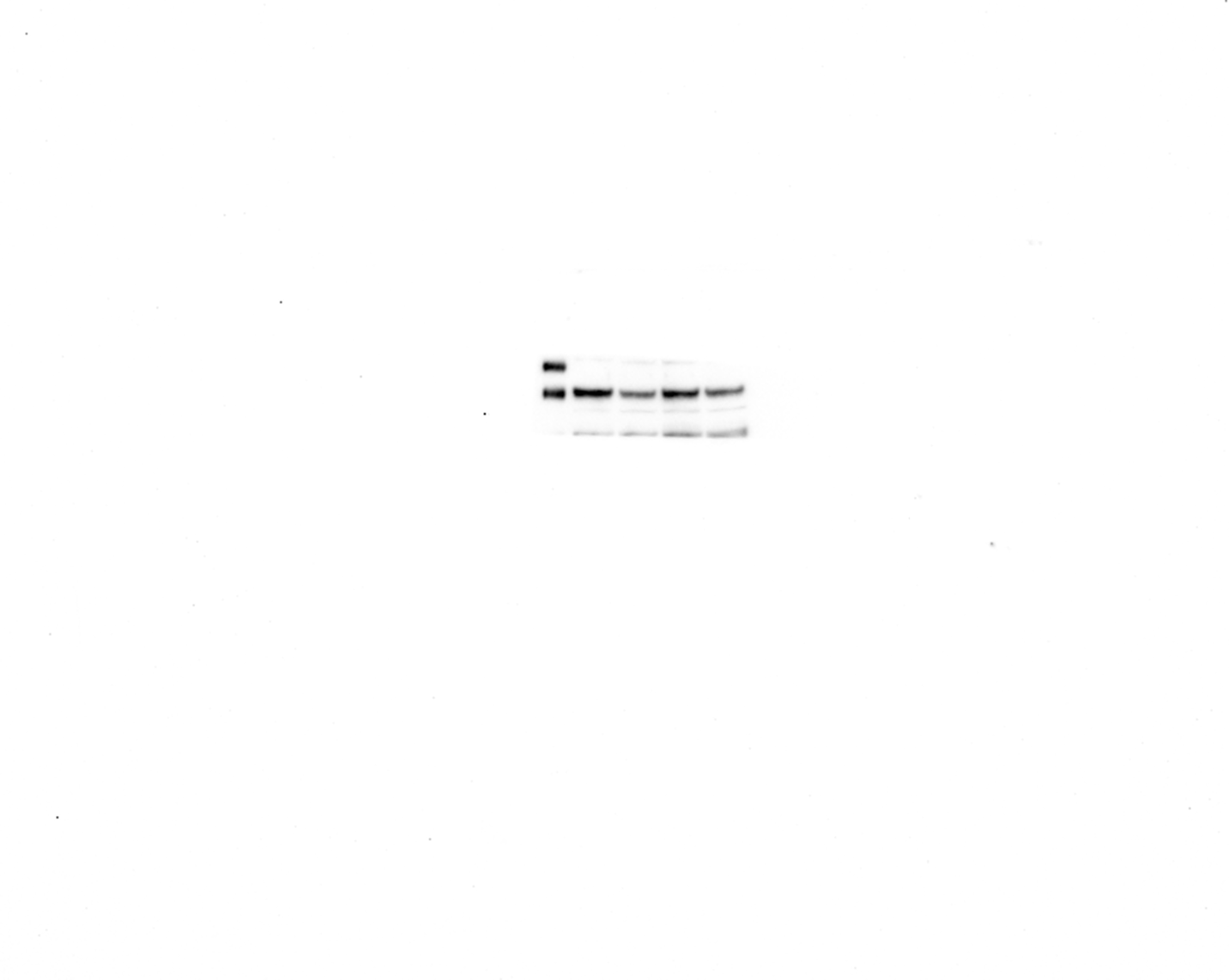


tubulin
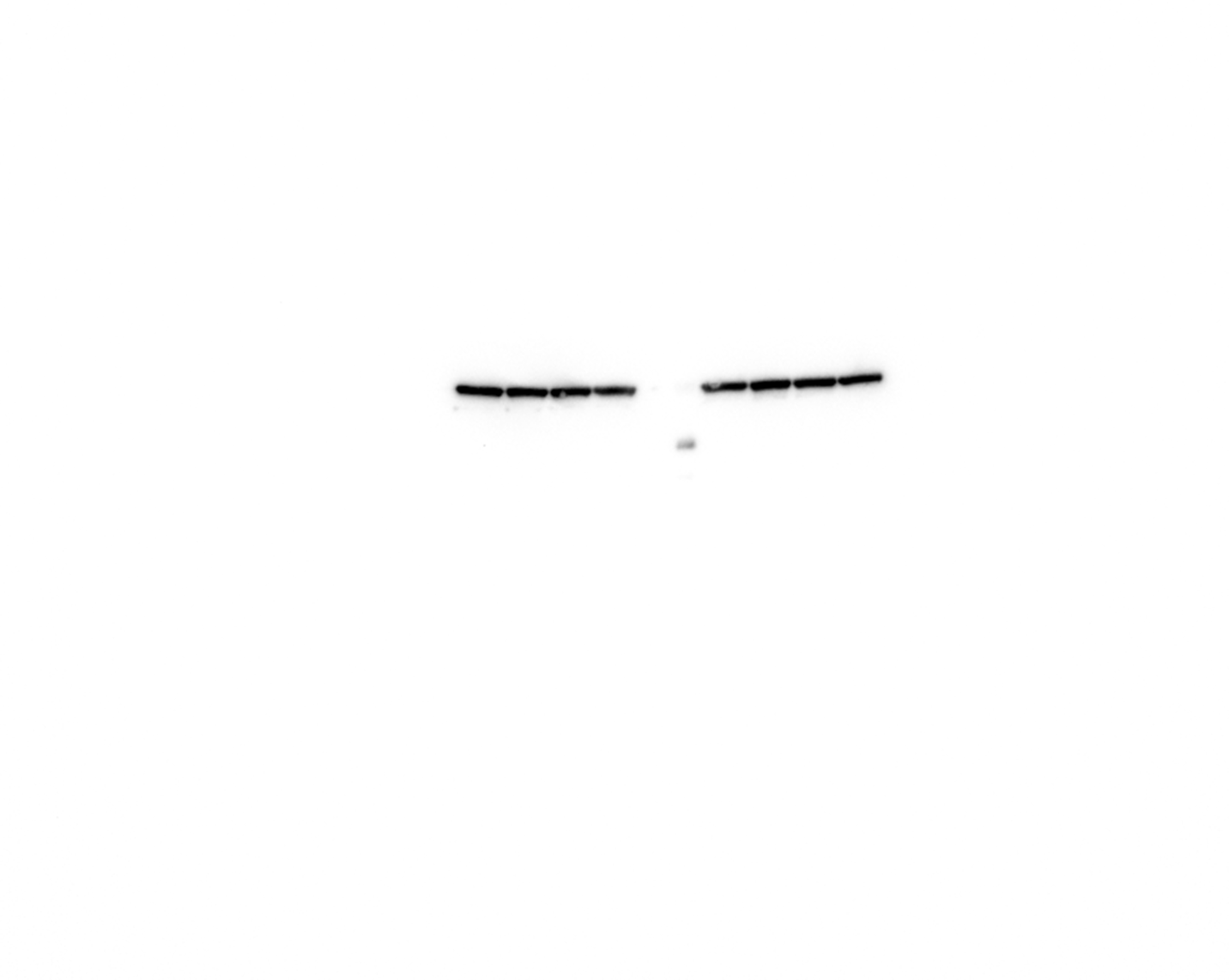


tYAP
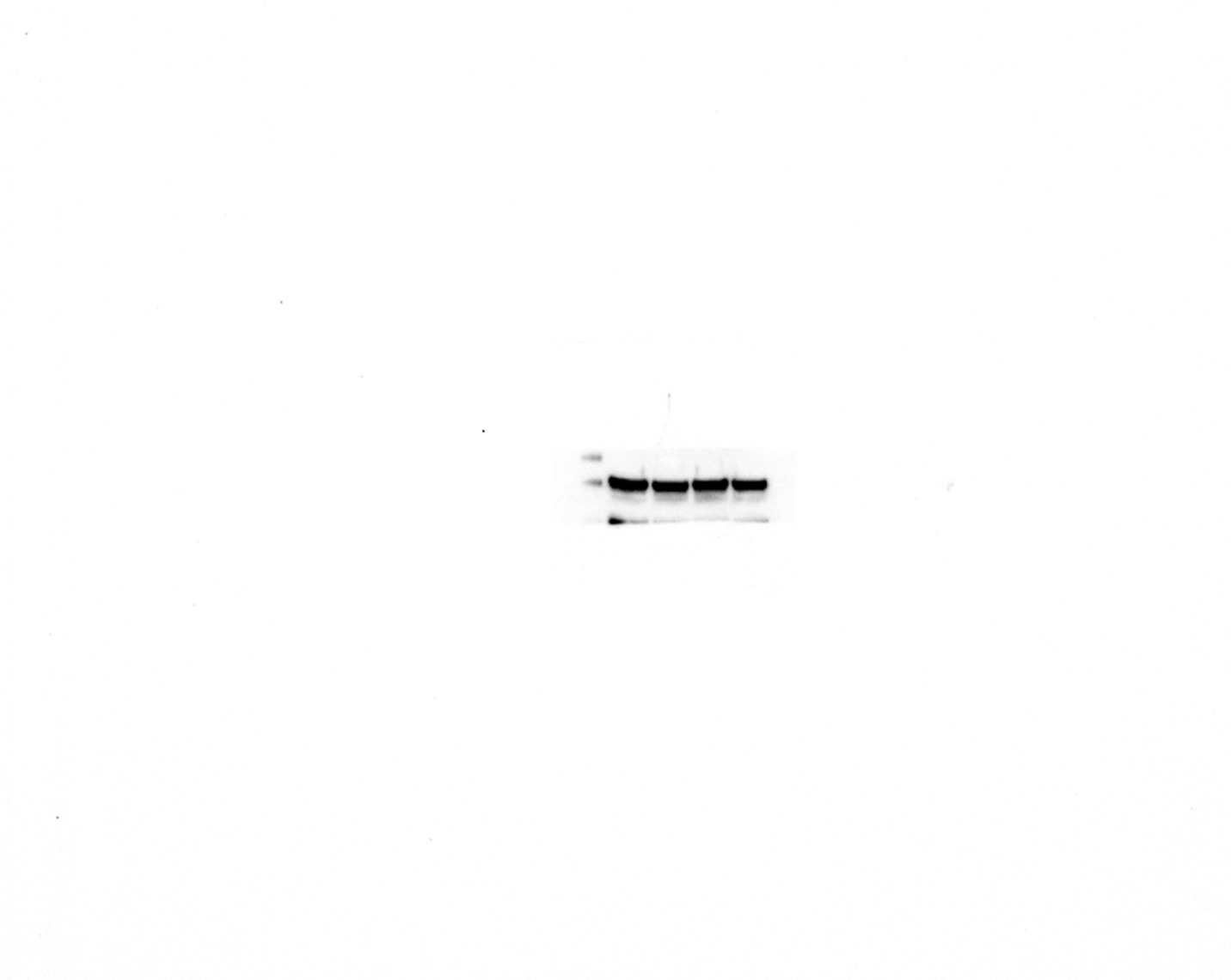


**Supplemental Figure 2A**- XMU utricle assay

pYAP
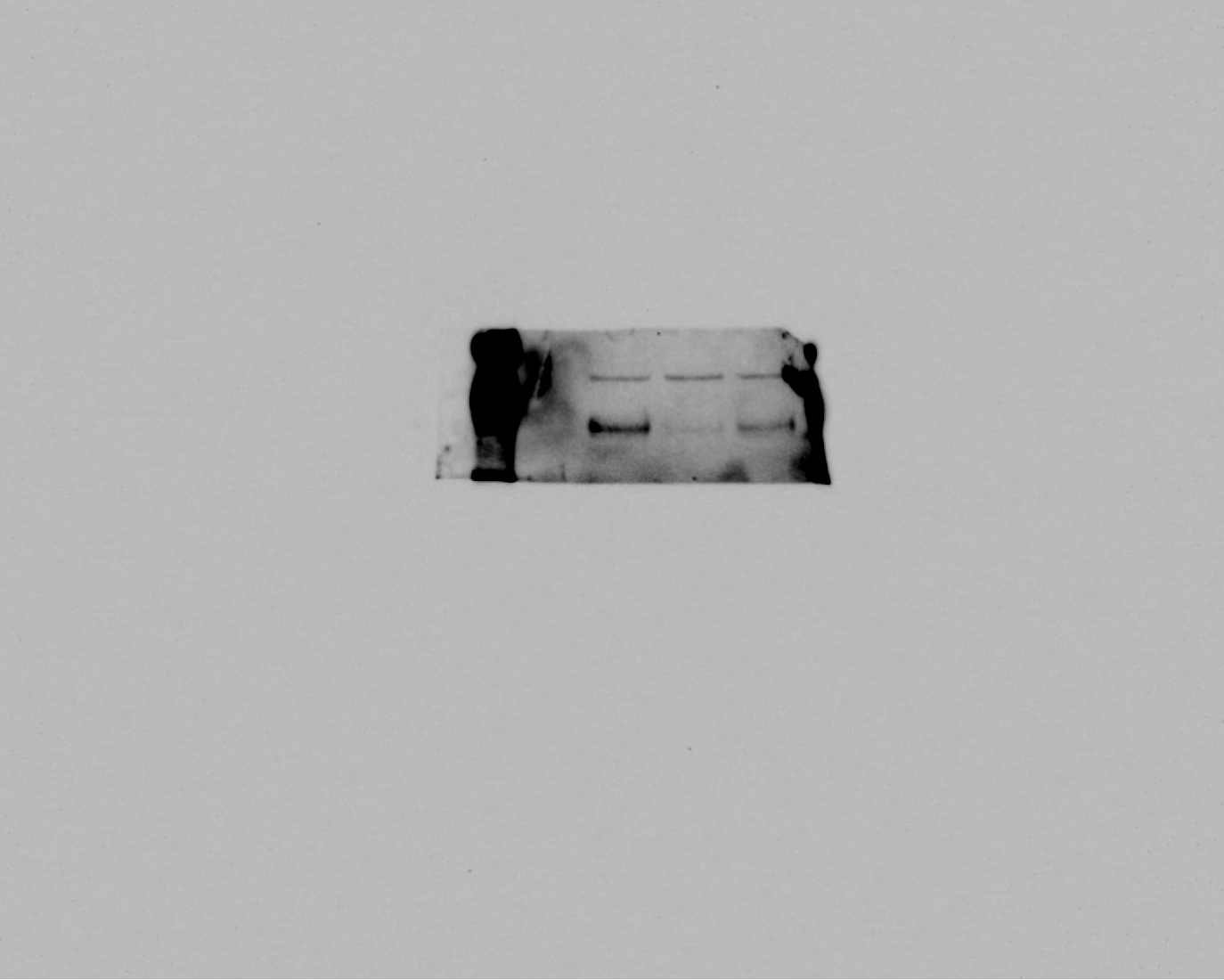


tYAP
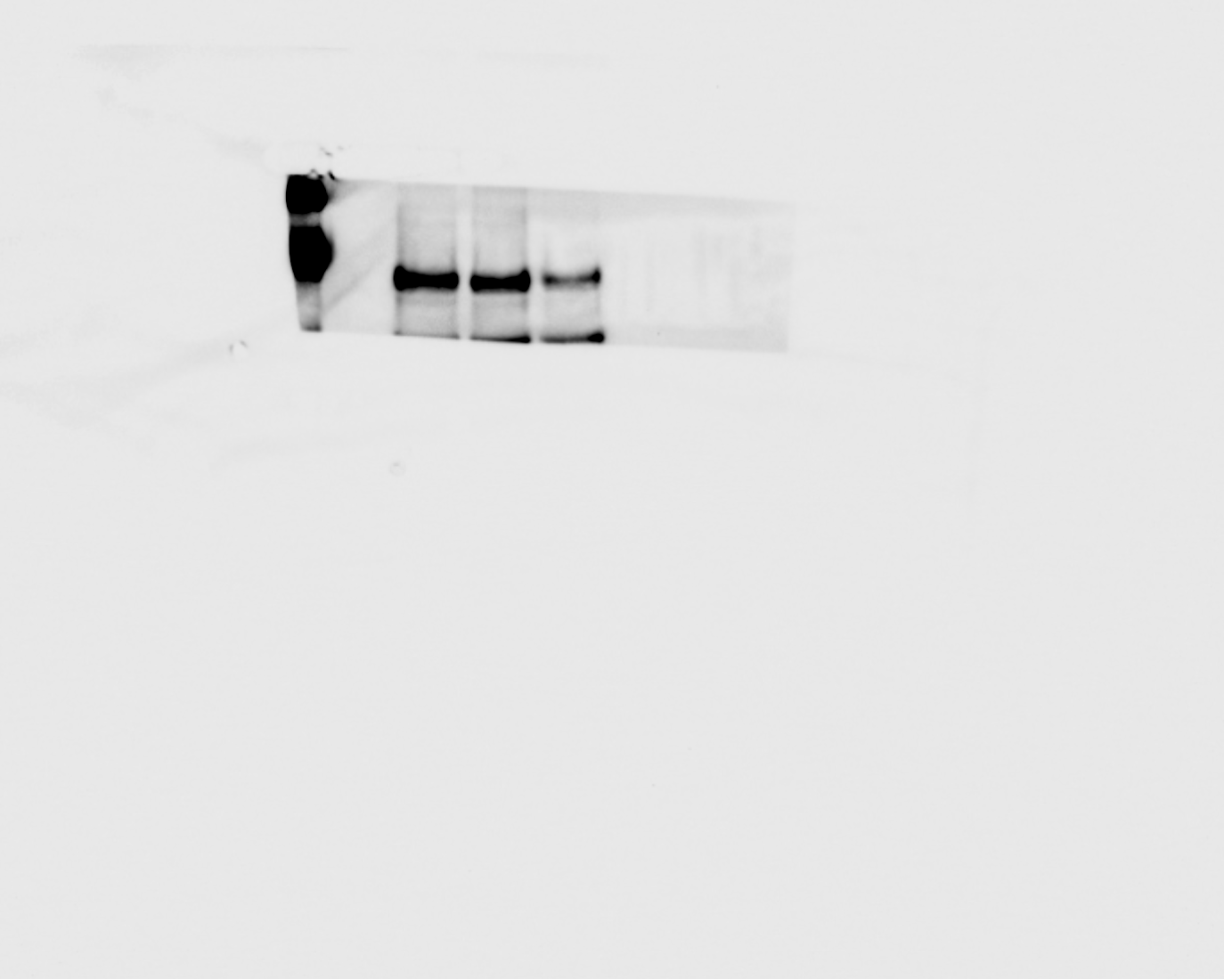


Tubulin
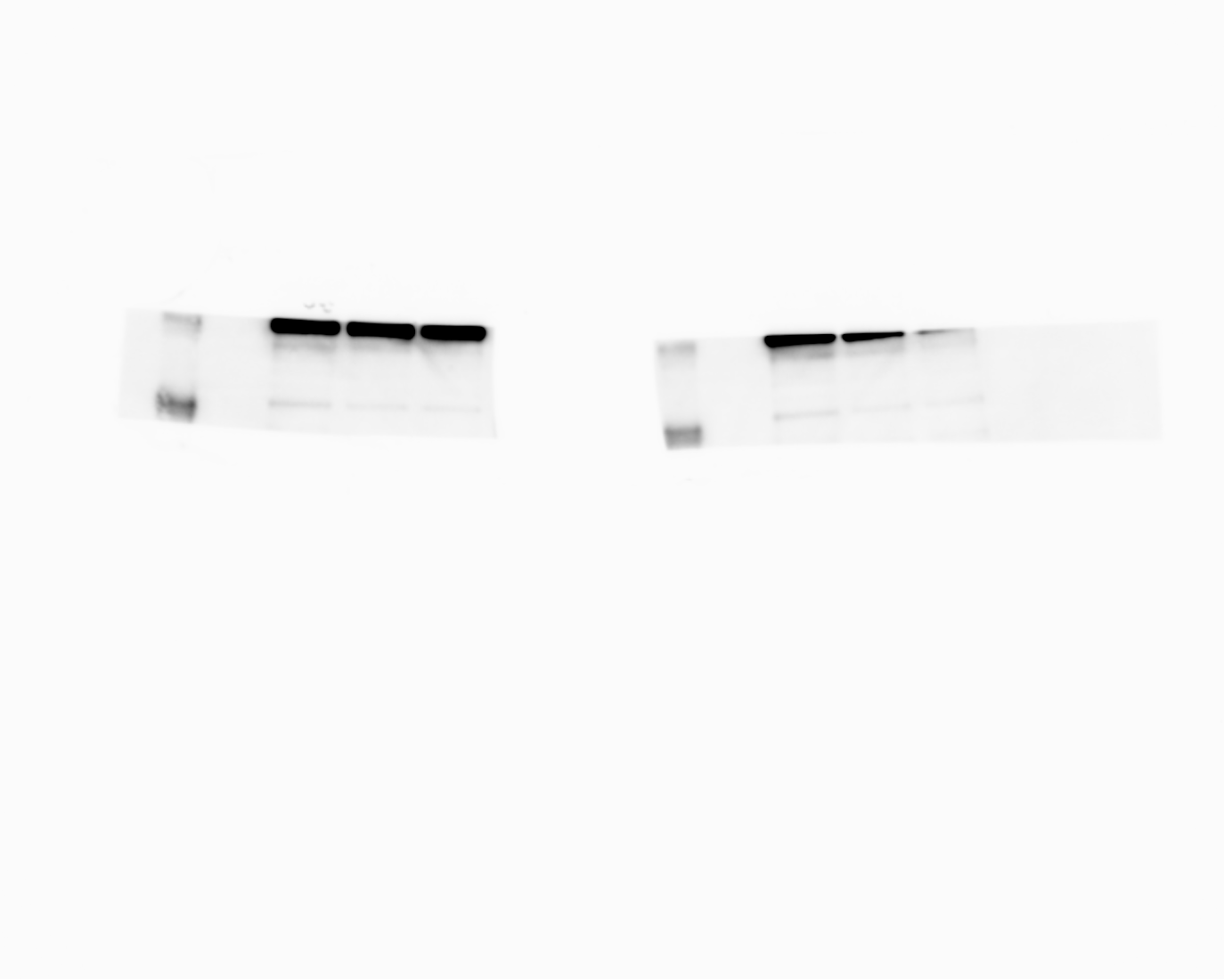

Supplement: Supplementary file 7 — Source Data [file 41467_2021_23395_MOESM7_ESM.zip › Source data-raw WB's.docx]
